# Supplementary material for: Age and market capitalization drive large price variations of cryptocurrencies
Source: Sci Rep. 2023 Mar 30;13:3351. doi: 10.1038/s41598-023-30431-3 (PMC10063656; doi:10.1038/s41598-023-30431-3)
Supplement: Supplementary file 1 — Supplementary Information. [file 41598_2023_30431_MOESM1_ESM.pdf]

Age and market capitalization drive large price variations of cryptocurrencies

Arthur A. B. Pessa[1,+], **Matjaž Perc**[2,3,4,5,6,*], **and Haroldo V. Ribeiro**[1,†]

[1]Departamento de Física, Universidade Estadual de Maringá - Maringá, PR 87020-900, Brazil
[2]Faculty of Natural Sciences and Mathematics, University of Maribor, Koroška cesta 160, 2000 Maribor, Slovenia
[3]Department of Medical Research, China Medical University Hospital, China Medical University, Taichung, Taiwan
[4]Alma Mater Europaea, Slovenska ulica 17, 2000 Maribor, Slovenia
[5]Complexity Science Hub Vienna, Josefstädterstraße 39, 1080 Vienna, Austria
[6]Department of Physics, Kyung Hee University, 26 Kyungheedae-ro, Dongdaemun-gu, Seoul, Republic of Korea
[+]email: arthur_pessa@hotmail.com
[*]email: matjaz.perc@gmail.com
[†]email: hvr@dfi.uem.br

Supplemental Materials

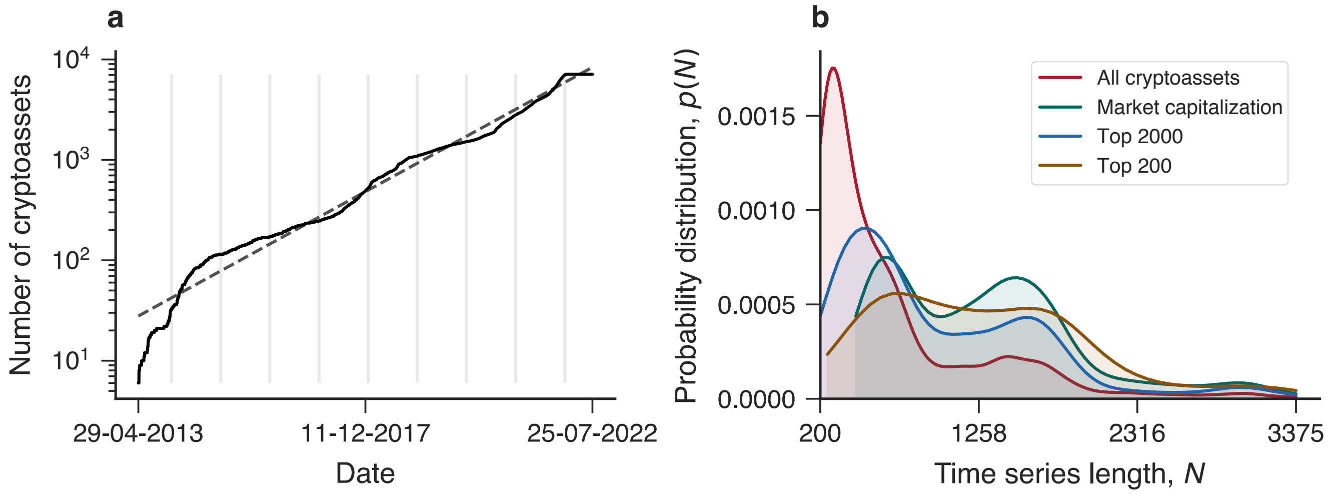

Figure S1. Growth in the number of cryptoassets listed on CoinMarketCap and their age distribution. (**a**) The continuous curve shows the accumulated number of cryptoassets listed on CoinMarketCap with more than 200 return observations on 25 July 2022. The gray vertical lines in the background indicate the years from 2014 to 2022, while the dashed line represents an exponential function adjusted to data. According to this exponential model, the number of cryptoassets is doubling every 1.1 year (every 410 days). Moreover, 50% of all cryptoassets in our database have their first price record after 30 April 2021. (**b**) Length distributions of return series in our database. The red line shows the distribution for all 7111 cryptocurrencies, for which the median length value is 446 observations. The green curve shows the distribution for the 2140 cryptocurrencies having more than 50 observations of market capitalization concomitantly to the time series of the power-law exponents. The median length of these time series is 1244 observations. The blue curve shows the distribution for the top 2000 cryptoassets by market capitalization. These time series have a median length equal to 741 observations. The brown curve shows the distribution for the top 200 cryptoassets by market capitalization. These time series have a median length equal to 1183 observations.

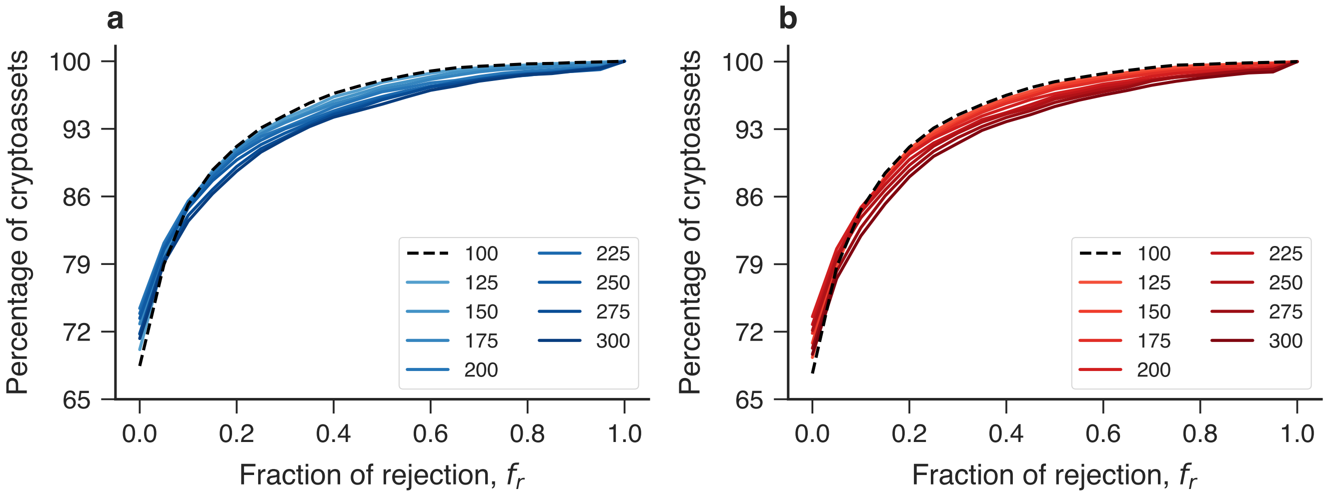

Figure S2. Robustness of the results of Fig. 2(a) against different numbers of initial observations in the expanding time window. Percentage of cryptoassets rejecting the power-law hypothesis for large (**a**) positive and (**b**) negative price returns in a given fraction of the weekly positions of the expanding time window (f_r) used to sample the return series. In both panels, the different curves show the results for initial observations ranging from 100 (dashed lines, exactly as reported in the main text) to 300 (colored curves).

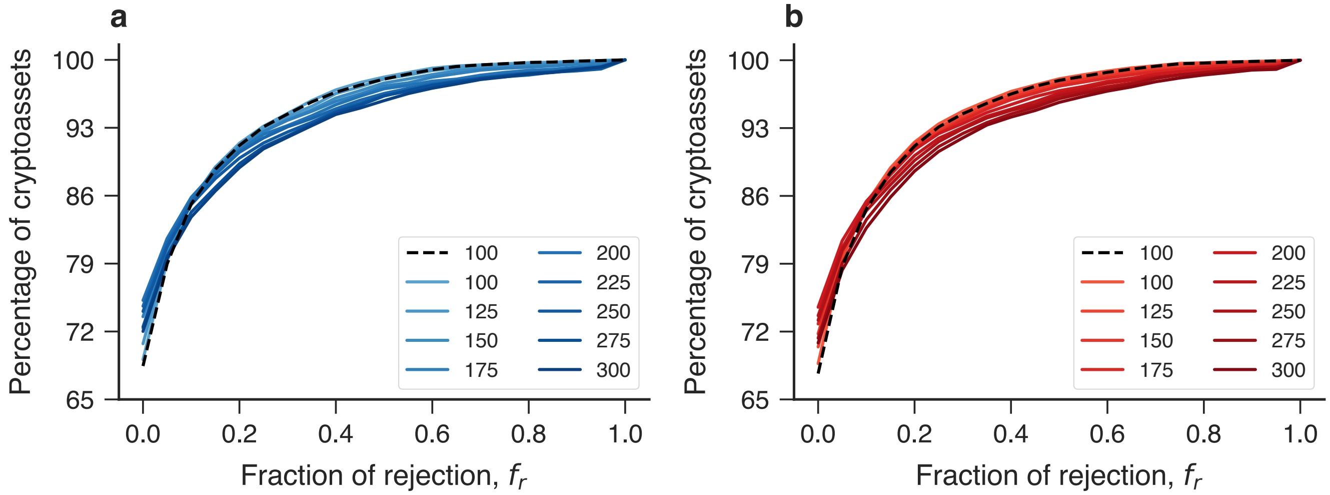

Figure S3. Robustness of the results of Fig. 2(a) against filtering out time series with sampling issues. Percentage of cryptoassets rejecting the power-law hypothesis for large (**a**) positive and (**b**) negative price returns in a given fraction of the weekly positions of the expanding time window (f_r) used to sample the return series. In both panels, the different curves show the results after entirely removing the data from cryptoassets with sampling issues and for initial observations ranging from 100 to 300 (colored curves). The dashed lines show the results as reported in the main text. Sampling issues refer to missing data and problems caused by prices of cryptoassets decreasing to zero.

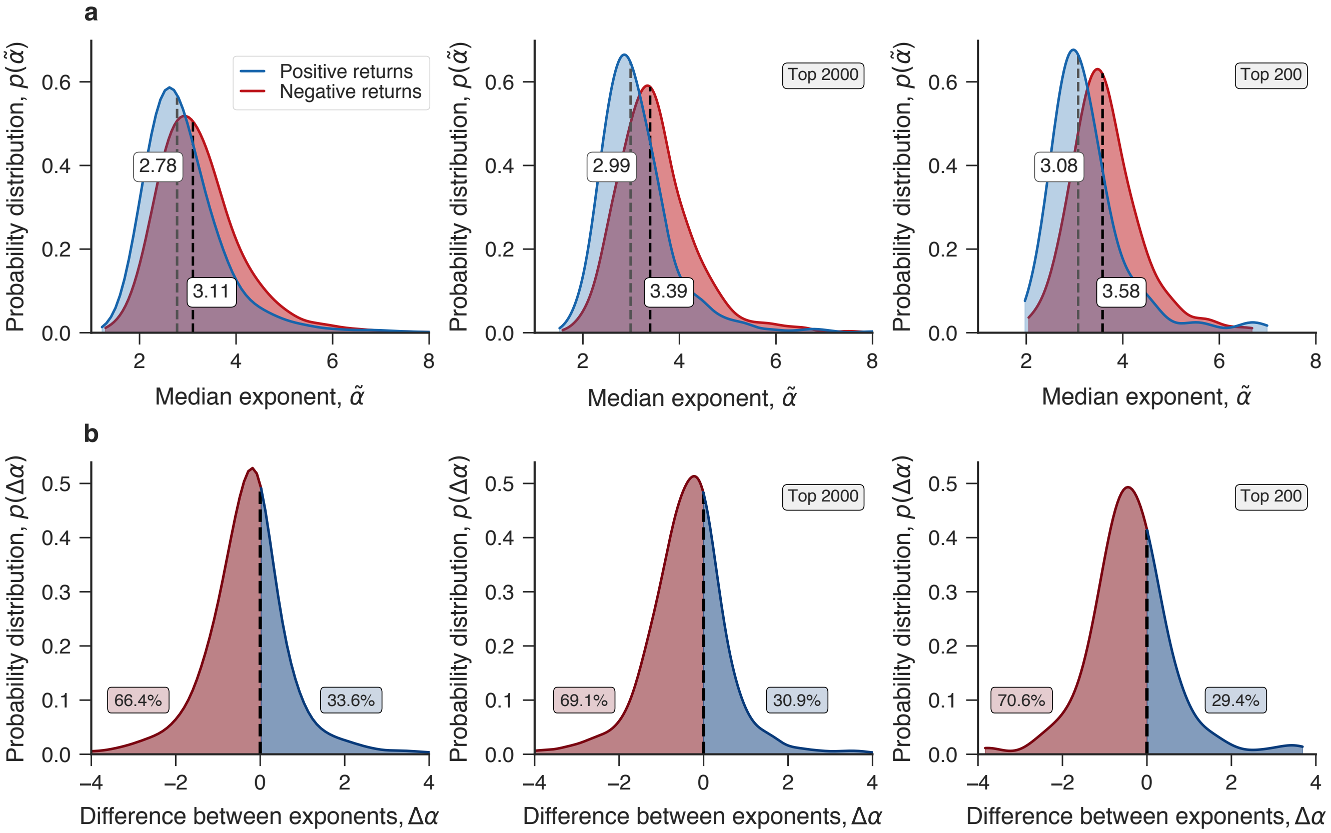

Figure S4. Probability distributions of the median power-law exponents associated with positive ($\tilde{\alpha}_+$) and negative ($\tilde{\alpha}_-$) returns and their asymmetric behavior quantified by the distributions of $\Delta\alpha = \tilde{\alpha}_+ - \tilde{\alpha}_-$. (**a**) Probability distributions obtained via kernel density estimation of the median values of the power-law exponents along the history of each digital currency. The blue curve shows the distribution of the median exponents related to positive returns ($\tilde{\alpha}_+$) and the red curve does the same for negative returns ($\tilde{\alpha}_-$). The medians of $\tilde{\alpha}_+$ and $\tilde{\alpha}_-$ are indicated by vertical dashed lines. These are the same distributions shown in Figs. 2(b)-(d) of the main text and were once again depicted here for comparison purposes. (**b**) Probability distributions of the differences between the median power-law exponents associated with positive and negative returns ($\Delta\alpha = \tilde{\alpha}_+ - \tilde{\alpha}_-$) for each cryptocurrency. The first column of panels depicts the results when considering data from all cryptocurrencies, while the second and third columns present the results for the top 2000 and top 200 cryptocurrencies by market capitalization, respectively.

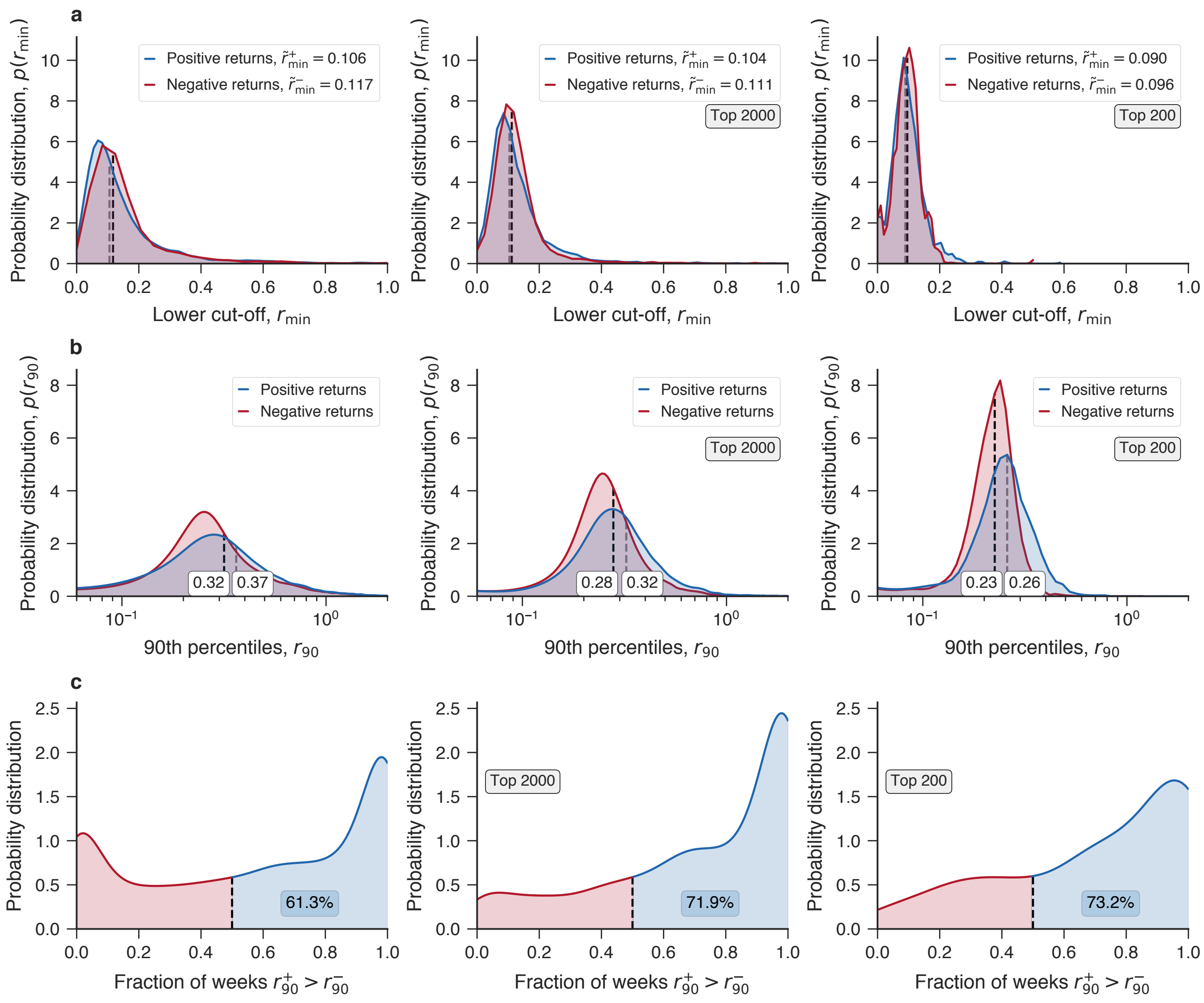

Figure S5. There is more probability mass in the positive tail than in the negative tail of price returns. (**a**) Probability distributions of the lower cut-offs ($r_{\min}$) obtained by applying the Clauset-Shalizi-Newman method to positive (blue) and negative (red) returns. The vertical dashed lines indicate the median values of $r_{\min}$ for positive and negative returns. (**b**) Probability distributions of 90th percentiles (r_{90}) estimated from the power-law models adjusted to positive (blue) and negative (red) returns. The vertical dashed lines indicate the median values of r_{90} for positive and negative returns. (**c**) Probability distributions of the fraction of weeks that r_{90} estimated from positive returns (r^{+}_{90}) is larger than r_{90} estimated from negative returns (r^{-}_{90}). This fraction is calculated only for weeks in which the power-law hypothesis is not rejected for both tails. The percentage of cryptoassets for which $r^{+}_{90} > r^{-}_{90}$ is shown in the panels. The first column of panels depicts the results when considering data from all cryptocurrencies, while the second and third columns present the results for the top 2000 and top 200 cryptocurrencies by market capitalization, respectively.

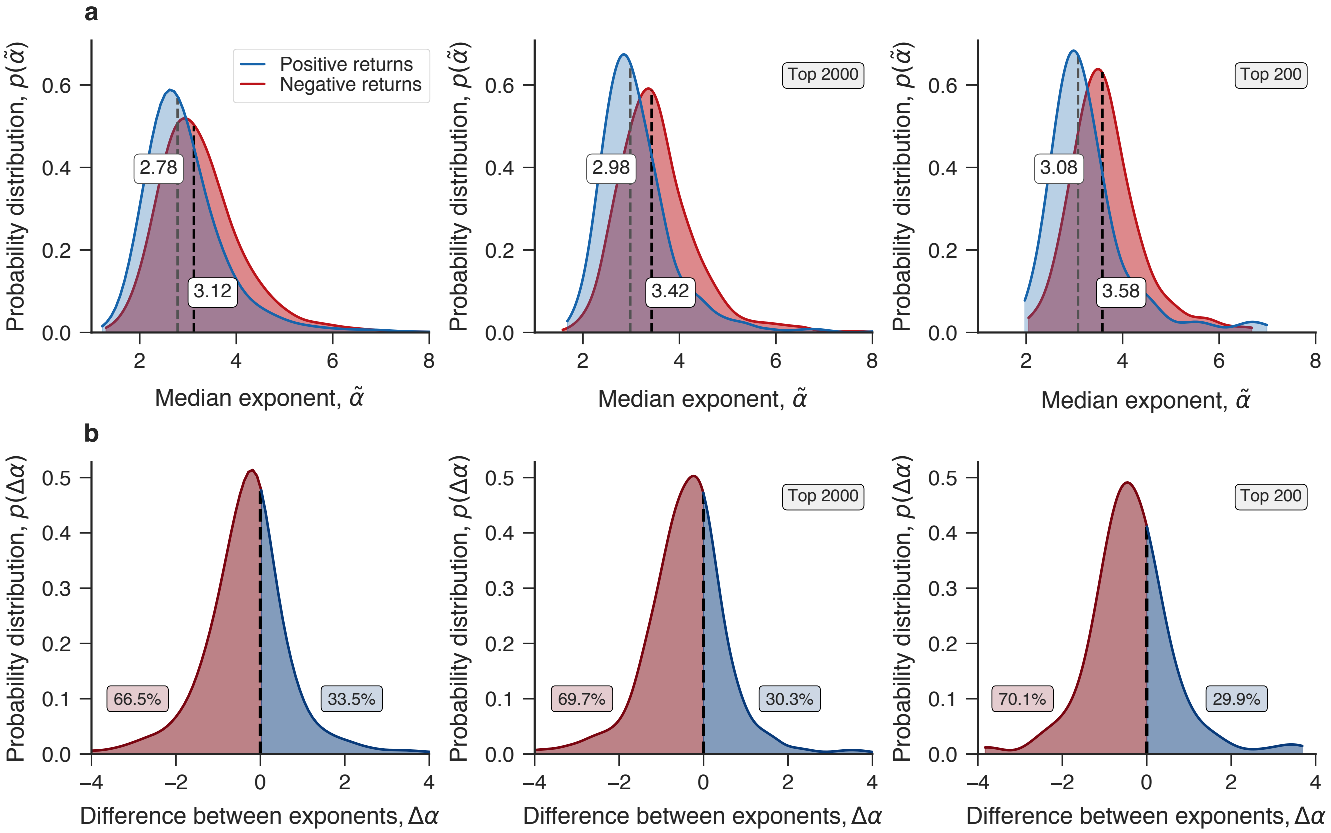

Figure S6. Robustness of the results of Fig. 2(b)-(d) against filtering out time series with sampling issues. Panels (**a**) and (**b**) show the same distributions of Fig. S4 but after filtering out all time series with sampling issues. Sampling issues refer to missing data and problems caused by prices of cryptoassets decreasing to zero. We note that these distributions barely change when considering only cryptocurrencies without any sampling issue. Indeed, the distributions in this figure are not significantly distinguishable from their counterparts in Fig. S4 (two-sample Kolmogorov-Smirnov test, $p > 0.05$).

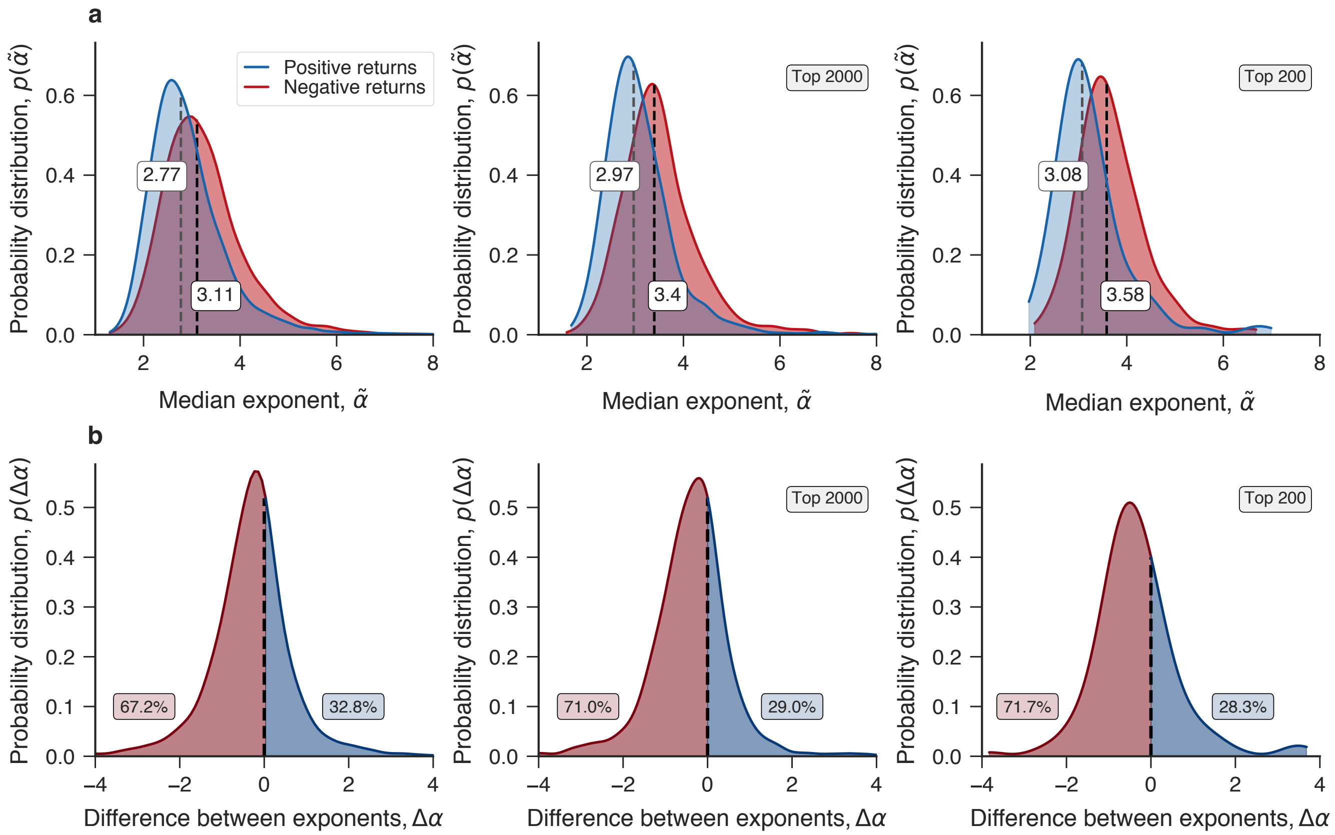

Figure S7. Robustness of the results of Fig. 2(b)-(d) against considering only cryptocurrencies with fraction of rejection $f_r < 0.1$. Panels (**a**) and (**b**) show the same distributions of Fig. S4 but after filtering out all time series of cryptocurrencies with fraction of rejections $f_r \geq 0.1$. As in the case related to sampling issues, we observe that these distributions barely change when considering only cryptocurrencies with $f_r < 0.1$. Indeed, the distributions in this figure are not significantly distinguishable from their counterparts in Fig. S4 (two-sample Kolmogorov-Smirnov test, $p > 0.05$).

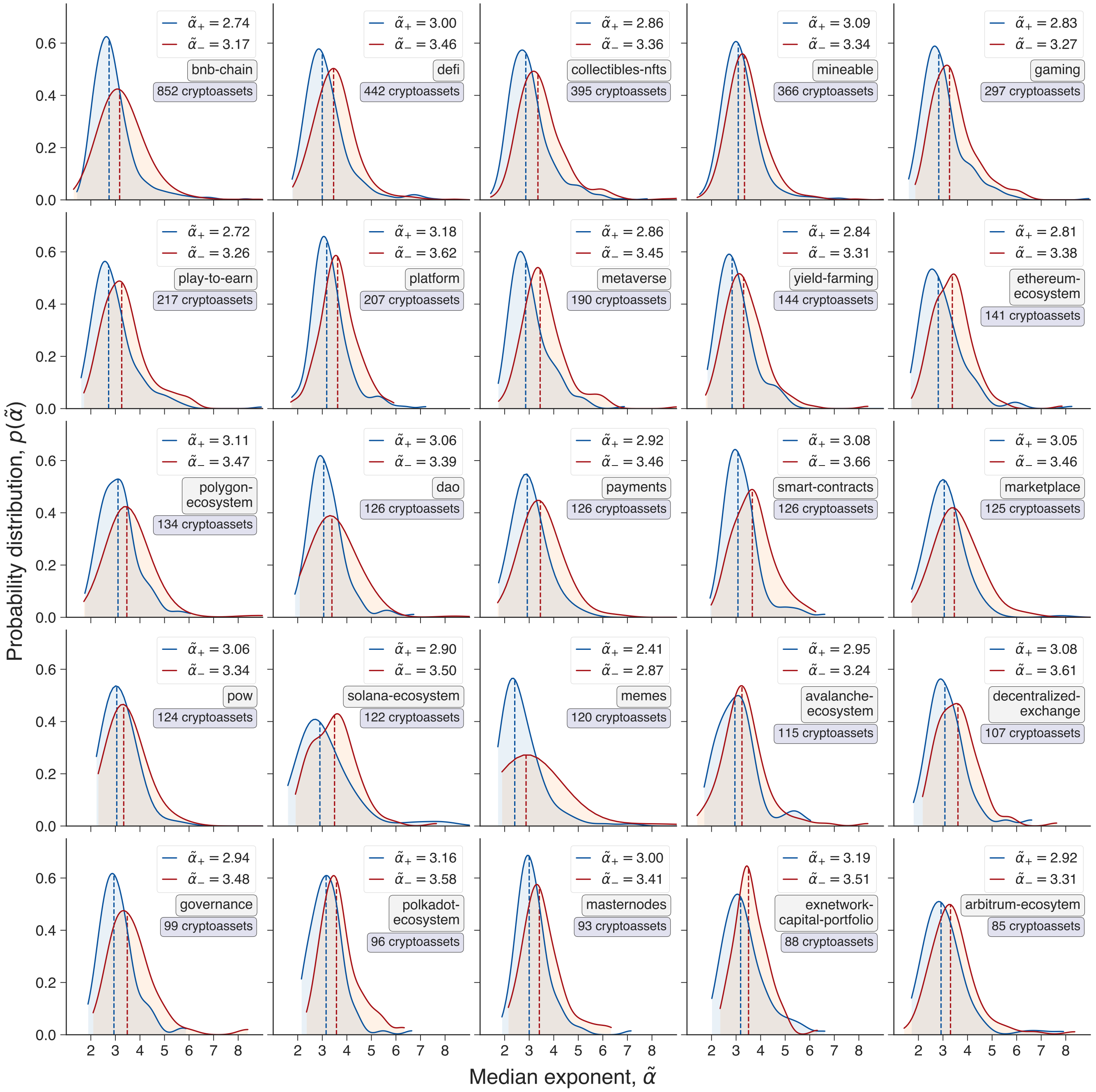

Figure S8. Probability distributions obtained via kernel density estimation of the median values of the power-law exponents along the history of each digital currency after grouping cryptoassets by the top 50 tags (indicated within panels). The blue curves show the distributions of the median exponents related to positive returns ($\tilde{\alpha}_+$) and the red curves do the same for negative returns ($\tilde{\alpha}_-$). The medians of $\tilde{\alpha}_+$ and $\tilde{\alpha}_-$ are indicated by vertical dashed lines. Results here refer to the top 25 tags (ranked by number of assets) and all pairs of distributions are significantly different from each other (two-sample Kolmogorov-Smirnov test, p-value ≤ 0.05).

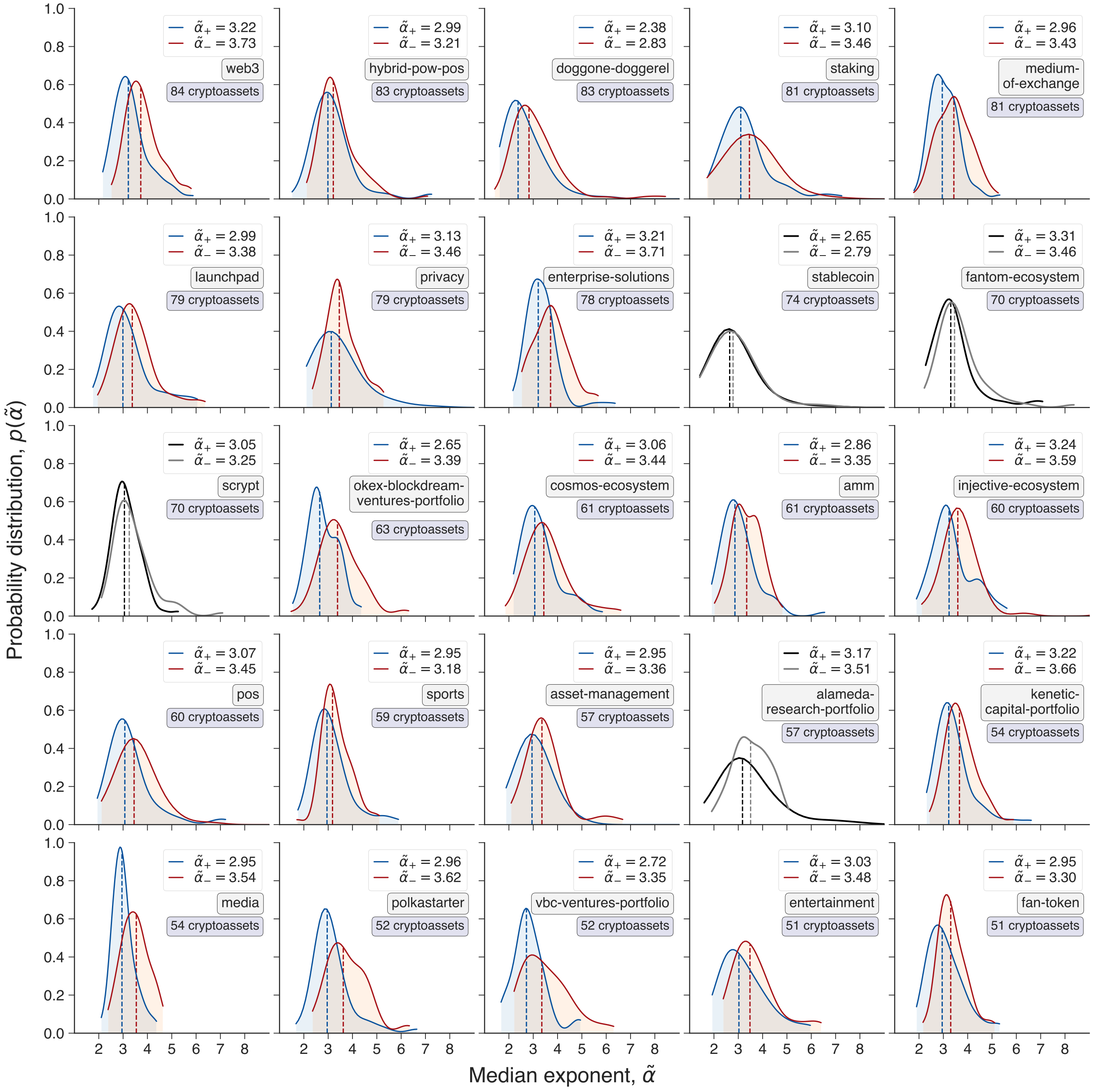

Figure S9. The same as Fig. S8 but for tags between the top 25 and top 50 (ranked by number of assets). Black and gray curves indicate pairs of distributions that are not significantly different from each other (two-sample Kolmogorov-Smirnov test, p-value > 0.05).

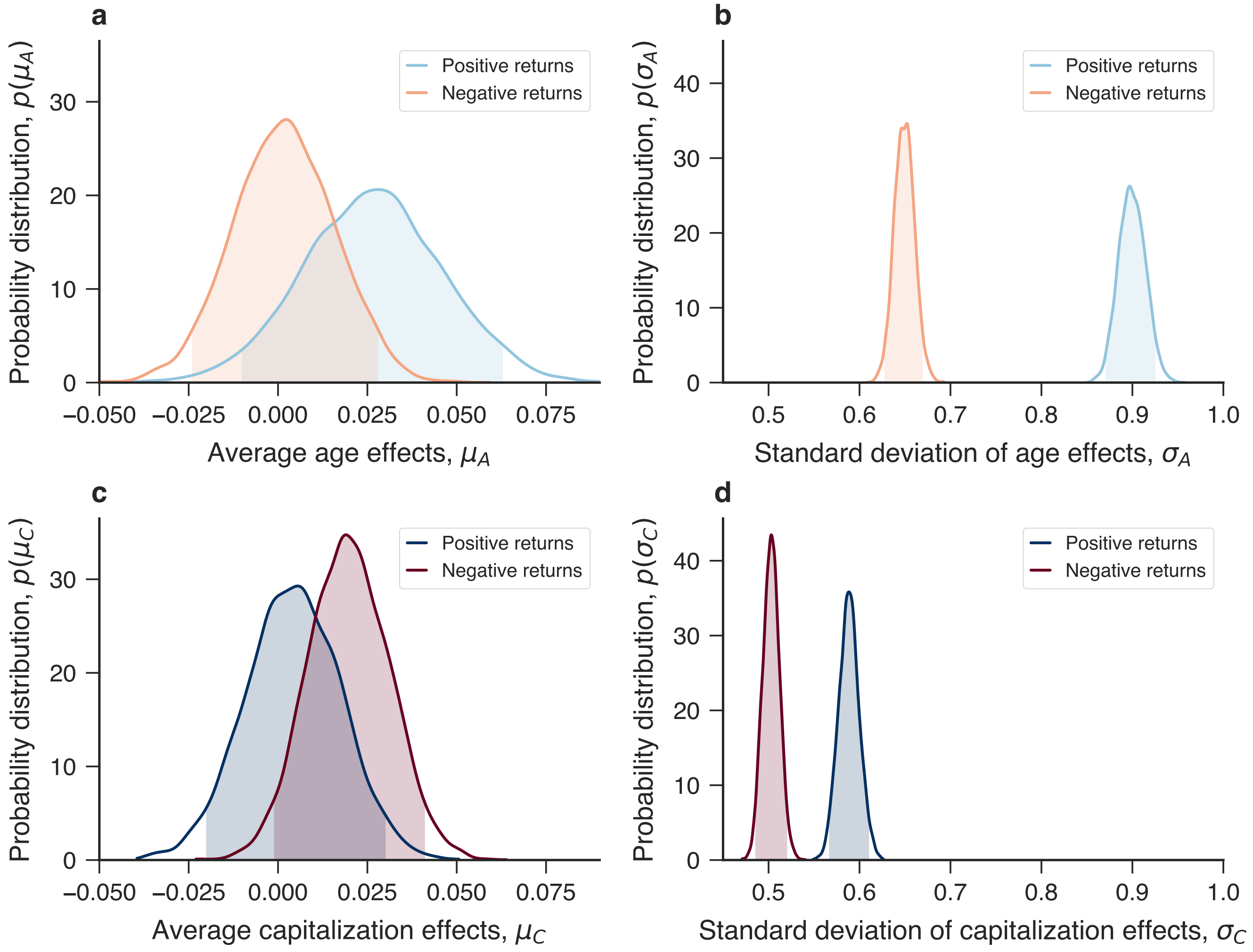

Figure S10. Overall effects of age and market capitalization on the evolution of the power-law exponents. (**a**) Posterior probability distributions for values of μ_A representing the overall effect of age on the evolution of the power-law exponents. (**b**) Posterior probability distributions for values of σ_A representing the standard deviation of the overall effect of age on the evolution of the power-law exponents. (**c**) Posterior probability distributions for values of μ_C representing the overall effect of market capitalization on the evolution of the power-law exponents. (**d**) Posterior probability distributions for values of σ_C representing the standard deviation of the overall effect of market capitalization on the evolution of the power-law exponents. In all panels, curves in blue shades refer to the power-law exponents associated with large positive price variations, while red shades refer to the exponents related to large negative price variations. The shaded regions below each distribution curve represent the 94% highest density intervals.

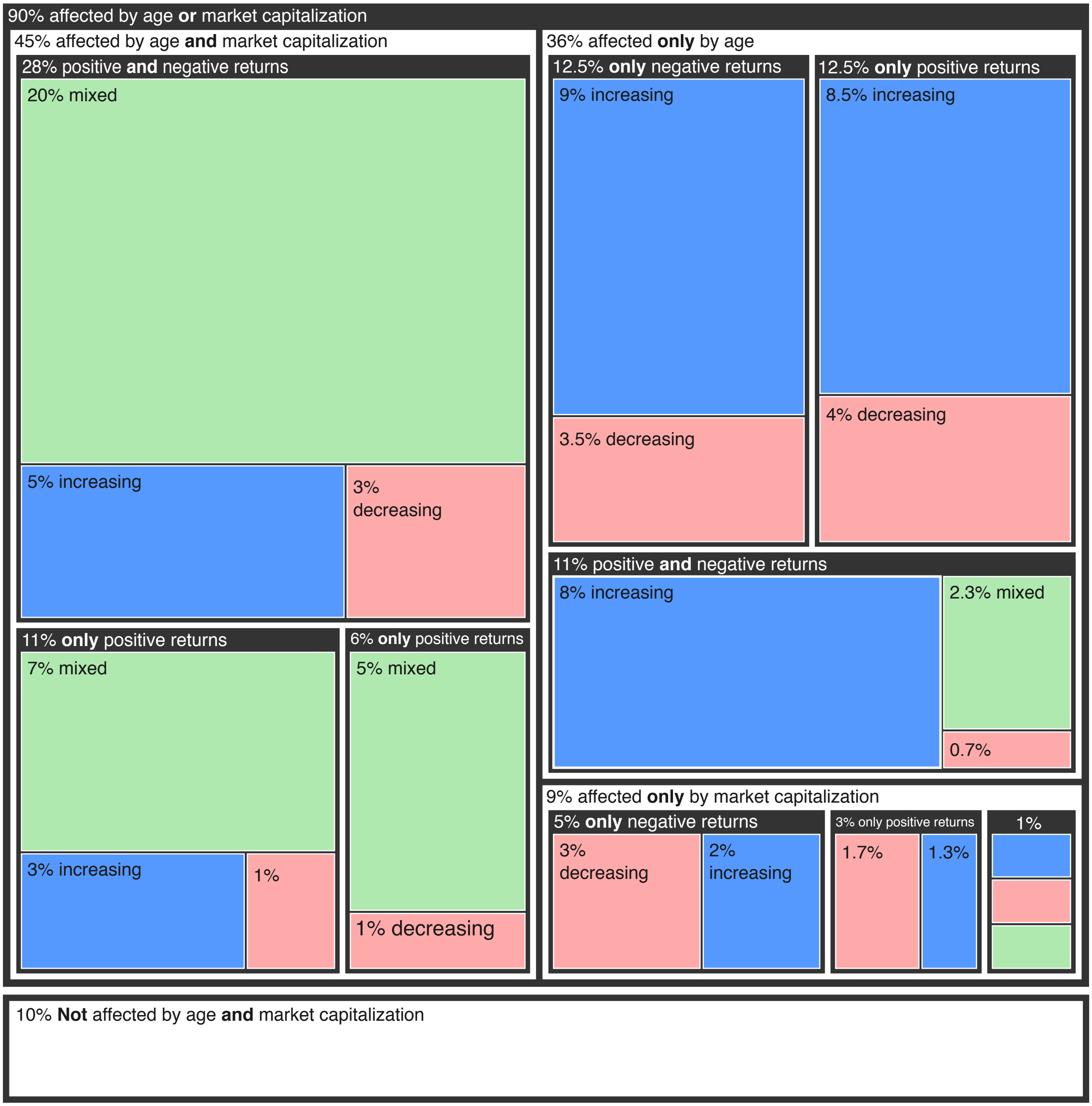

Figure S11. Summary of the effects of age and market capitalization on power-law exponents of the top 200 cryptocurrencies. Hierarchical visualization or a tree map of the possible effects of age and market capitalization on the power-law exponents. The first level (two outermost rectangles) separates cryptocurrencies that are affected by age or market capitalization (90%) from those unaffected by any of these quantities (10%). Cryptocurrencies affected by age or market capitalization are classified as those simultaneously affected by both quantities (45%), those affected only by age (36%), and those affected only by market capitalization (9%). Each of the previous three levels is further classified regarding whether both positive and negative returns are simultaneously affected or whether the effect involves only positive or only negative returns. Finally, the former levels are classified regarding whether the power-law exponents increase, decrease or have a mixed trend with the predictive variables. Overall, 35% of the associations are classified as mixed trends (green rectangles), 37% are increasing trends (blue rectangles), and 18% are decreasing trends (red rectangles).

Cryptocurrency	Ticker	Rank	Return sign
Monsta Infinite	MONI	1141	Negative
Feellike	FLL	2528	Negative
DAO Invest	VEST	3934	Positive
Gorilla Diamond	GDT	6011	Negative
Scientia	SCIE	7842	Positive
Future-Cash Digital	FCD	8640	Negative
Intersola	ISOLA	8961	Positive
NinjaFloki	NJF	9506	Negative
The Silent Sea	TSSEA	9533	Positive

Table S1. List of cryptocurrencies for which the power-law description of large price movements is always rejected.

Model predictors	Return sign	WAIC (standard error)	PSIS-LOO (standard error)
Age and market capitalization	Positive	**-322134 (7400)**	**-320681 (6965)**
Only age	Positive	-337340 (6843)	-336284 (6526)
Only market capitalization	Positive	-377644 (7530)	-377086 (7364)
Age and market capitalization	Negative	**-331334 (4573)**	**-330930 (4524)**
Only age	Negative	-343971 (4225)	-343762 (4198)
Only market capitalization	Negative	-377939 (4108)	-377855 (4096)

Table S2. Comparison of the goodness of fit among the models describing the power-law exponents as a function of age and market capitalization (Eq. 3 in the main text), only as a function of age, and only as a function of market capitalization. We have estimated the values of the Widely Applicable (or Watanabe-Akaike) Information Criterion (WAIC) and Pareto Smoothed Importance Sampling Leave-One-Out cross-validation (PSIS-LOO) as well as their standard errors. WAIC represents an estimate of expected out-of-sample-prediction error and PSIS-LOO represents an estimate of expected out-of-sample-prediction error for each Bayesian model. The smaller the values of the WAIC and PSIS-LOO coefficients, the better the quality of fit. Thus, the models using age and market capitalization for describing the dynamics of the power-law exponents associated with positive and negative returns represent the best description for our data.
